# Fracture Properties of High-Performance High-Ductility Alkali-Activated Concrete Under Thermo-Mechanical Coupling: Effect of Fiber Hybrid Ratio

**DOI:** 10.3390/polym17192568

**Published:** 2025-09-23

**Authors:** Tao Huang, Bo-Yuan Huang, Zhi-Feng Zhan, Yu-Wen Huang, Lei Wang, Meng Li, Zhi-Hong Xie, Jian-Fa Li, Jia-Ying Su

**Affiliations:** 1 Zhanjiang Power Supply Bureau, Guangdong Power Grid Co., Ltd., Zhanjiang 524200, China; 13828257686@163.com (T.H.); 18022660667@163.com (B.-Y.H.); 13600381851@163.com (Z.-F.Z.); huangyuwen1732@126.com (Y.-W.H.); 13670978260@163.com (L.W.); 13828220884@163.com (M.L.); 2 School of Future Transportation, Guangzhou Maritime University, Guangzhou 510060, China; hz.xzh@163.com; 3 School of Civil and Transportation Engineering, Guangdong University of Technology, Guangzhou 510006, China; lijianfahz@foxmail.com

**Keywords:** high-ductility, alkali-activated composites, fracture properties, fiber hybrid ratio, coupled temperature

## Abstract

This study investigated the fracture behavior of high-ductility alkali-activated composites (HDAACs) under thermo-mechanical coupling. Fracture tests were conducted on hybrid polypropylene/polyethylene (PP/PE) fiber-HDAAC with varying PP fiber replacement ratios (0%, 25%, and 50%) and coupled temperatures (0 °C, 30 °C, 70 °C, 100 °C, and 150 °C). The fracture mechanisms were analyzed through failure modes, mode I fracture energy (*G*_F_), and the J-integral method. The results showed that below 100 °C, specimens exhibited ductile failure with a main crack along the notch accompanied by stable matrix cracking, with *G*_F_ peaking at 16.47 kJ/m^2^. At 150 °C, fiber melting led to a reduction in *G*_F_ to 2.01 kJ/m^2^. Initial cracking energy (*J*_IC_ ≈ 0.1 kJ/m^2^) remained stable, while unstable fracture energy (*J*_IF_) peaked at 70 °C and dropped sharply at 150 °C. The energy consumed by matrix cracking showed (*J*_m_) a similar trend to that consumed by fiber pull-out and fracture (*J*_b_), with *J*_m_/*J*_C_ = 0.4–0.5. Higher PP replacement reduced both *J*_m_ and *J*_b_. The fracture behavior differences under thermo-mechanical coupling versus post-heating were mainly due to fiber exposure timing. This study highlights the critical influence of thermo-mechanical coupling on HDAAC fracture mechanisms, offering guidance for designing HDAACs for high-temperature applications.

## 1. Introduction

Alkali-activated concrete (AAC) has emerged as a low-carbon alternative to traditional Portland cement concrete because of its favorable durability and reduced embodied carbon compared with conventional binders [1,2]. When combined with engineered cementitious composite (ECC) design principles, AAC can be formulated into a type of polymer fiber-reinforced composite, high-ductility AAC (HDAAC). HDAAC exhibits multiple cracking and steady-state cracking behavior under tension, delivering substantially enhanced tensile deformability compared with conventional concrete [3,4,5,6,7]. However, high-performance polymer fibers, like polyvinyl alcohol (PVA), polyethylene (PE), and polyethylene terephthalate fibers (PETs), are typically needed to ensure adequate fiber-bridging capacity [8,9,10,11]. These fibers can represent 50–80% of total material costs [11,12,13]. For instance, Kuraray Co., Ltd. (Tokyo, Japan) in Japan holds a monopoly on surface coating technology for PVA fibers, which further limits large-scale use due to the construction industry’s sensitivity to material costs.

To reduce costs, fiber hybridization offers a practical solution. As reported, Alrefaei and Dai [14] incorporated PE fibers and steel fibers in HDAAC, realizing the simultaneous improvement of compressive strength and ultimate tensile strain. Pakravan et al. [15] also demonstrated an increased ultimate tensile strain up to 33% and 148%, respectively, through the replacement of 20 vol.% of polypropylene (PP) fiber and low-modulus PVA fiber. PP fiber is a low-modulus and more affordable option, offering a cost-effective alternative to expensive fibers like PE and PVA fibers. It can significantly lower production expenses while maintaining the mechanical performance of HDAAC to some extent [16]. However, substituting PP for PE fibers may reduce the fiber-bridging efficiency and tensile performance due to a lower strength and weaker matrix bonding [15,16,17,18,19]. The careful adjustment of fiber ratios is therefore essential to balance cost-effectiveness and mechanical performance.

Furthermore, existing relevant studies have mostly focused on the basic mechanical properties under ambient temperature [20,21], while the studies regarding the fracture performance of HDAAC are rarely investigated and reported. HDAAC’s fracture process involves the formation of numerous microcracks within a fracture process zone and high energy dissipation. Thus, it has led to the inapplicability of classical models like the double-K fracture model [22]. Moreover, high service temperatures are common in residential, industrial, and infrastructure applications, where surfaces can reach 60–200 °C [23,24]. When HDAAC is applied in these environments, both the alkali-activated matrix and the fibers may be exposed to temperatures up to 200 °C, often alongside mechanical loading. While AAC matrices demonstrate excellent thermal resistance [25,26], PE and PP fibers experience reduced tensile strength and increased elongation at elevated temperatures [27]. Consequently, HDAAC’s fracture behavior under thermo-mechanical coupling may differ significantly from post-heating residual responses.

Although few studies have examined the fracture behavior of HDAAC under thermo-mechanical coupling, some studies have explored its residual mechanical properties after high-temperature exposure. These studies suggested that moderate heating could enhance compressive strength by promoting the further activation of fly ash (FA) particles [24]. However, at higher temperatures, compressive strength declined due to fiber melting, which led to pore formation, microcracking, and matrix void growth [28,29]. Moreover, the tensile and flexural properties of HDAAC were reported to be sensitive to temperature because the mechanical behavior of both the matrix and fibers changes as temperature increased [30]. These findings highlight that the combined effects of heat and mechanical loading—particularly on the matrix, fibers, and their interface—are key to understanding how HDAAC performance is enhanced or degraded. As such, the fracture behavior of HDAAC under thermo-mechanical coupling likely differs significantly from its post-heating response and better represents actual service conditions. In most existing studies, specimens were tested after being cooled from a target temperature. However, this approach did not accurately reflect real service conditions, where HDAAC were often subjected to simultaneous loading and high temperatures. Some researchers compared residual specimens (tested after cooling) with quasi in situ specimens (tested while still hot), finding that the latter exhibited a lower tensile strength. This difference was ascribed to a reduced fiber–matrix bond strength at elevated temperatures [31]. For example, Constâncio Trindade et al. [32] observed a greater strength loss in specimens tested while hot. In summary, far less attention has been given to the fracture behavior of HDAAC under thermo-mechanical coupling, where heat and load act simultaneously.

This study experimentally investigated the fracture behavior of hybrid PP/PE-HDAAC under thermo-mechanical coupling. The key variables included fiber hybrid ratios (0%, 25%, and 50%) and coupled temperatures (0 °C, 30 °C, 70 °C, 100 °C, and 150 °C). Using three-point bending fracture tests, the fracture failure modes of hybrid PP/PE-HDAAC under combined thermal and mechanical loading were analyzed. The evolution of load–displacement (*P-δ*) and load–crack mouth opening displacement (*P*-*CMOD*) curves was examined to characterize fracture behavior. To evaluate fracture deformation capacity, mode I fracture energy (*G*_F_) was calculated. Importantly, from an energy perspective, the J-integral method was applied to analyze energy dissipation during crack initiation, instability, and final fracture. These results provide new insights into HDAAC fracture mechanisms and offer engineering guidance for its practical application and sustainable development in high-temperature service environments.

## 2. Materials and Methods

### 2.1. Materials

The raw materials used in HDAAC are as follows: Class F FA and S105-grade ground granulated blast furnace slag (GGBFS) served as precursors for the alkali-activated binder. Quartz sand (QS) with a particle size of 76–150 µm was used as the fine aggregate. The alkali activator consisted of a 14 mol/L sodium hydroxide solution and sodium silicate with a modulus of 2.25. Ultra-high-molecular-weight polyethylene (UHMWPE) fibers (referred to as PE fibers) and polypropylene (PP) fibers were incorporated as reinforcement. Barium chloride (BaCl_2_) was added as a retarder at 1 wt% of the binder mass.

**Figure 1:**
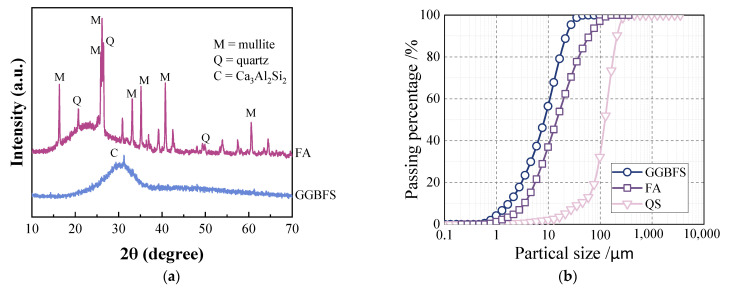
Physical and chemical property parameters of raw materials: (**a**) XRD patterns of FA and GGBS; (**b**) particle size distribution of raw materials.

The XRD patterns of FA and GGBS are shown in Figure 1a, while the particle size distributions of the precursors and fine aggregates are presented in Figure 1b. The chemical compositions of GGBS and FA are listed in Table 1. The physical properties of PE and PP fibers are detailed in Table 2.

Notably, the alkali activator solution (AAS) was prepared by mixing sodium silicate (SS) solution, with a modulus of 2.25, and 10 mol/L sodium hydroxide (SH) solution at a mass ratio of 2:1. The SS solution that has a composition ratio of SiO_2_:Na_2_O:H_2_O is 29.99:13.75:56.26 by mass. Sodium silicate was supplied by Yourui Refractory Materials Co., Ltd. (Jiaxing, China), and both SH (96% purity) and BaCl_2_ were provided by Xilong Scientific Co., Ltd. (Shantou, China).

Table 3 presents the mix proportions used in this study. The mix design was refined from the proportions proposed by Wang et al. [33], as shown in Table 3. The mass ratio of FA to GGBFS was fixed at 7:3. The quantities of QS, AAS, and BaCl_2_ were determined as ratios to the binder mass, specifically 0.2, 0.4, and 0.01, respectively. The total fiber content constituted 2% of the HDAAC volume fraction, with specimen group R25 indicating a 25% PP fiber replacement ratio, and other groups following analogous nomenclature. The fiber volume of HDAAC is generally less than 2 vol%, which has been reported frequently in previous studies [34,35]. In addition, only the PE–PP hybrid ratio was varied, ensuring that changes in mechanical response could be attributed to hybridization rather than differences in fiber content.

### 2.2. Specimen Preparation

The hybrid PE/PP-HDAAC specimens were prepared using a planetary mortar mixer. FA, GGBFS, QS, and BaCl_2_ were first dry-mixed at 75 r/min for 2 min. The cooled alkali solution and water were then added gradually while mixing at 75 r/min for 1 min, followed by mixing at 135 r/min for another min to ensure complete reaction with the alkaline activator. Fibers were then slowly sprinkled into the fresh matrix under continuous stirring (75 r/min for 2 min and 135 r/min for more 1 min) to prevent clumping and to promote uniform dispersion throughout the mixture. Given the relatively low total fiber volume fraction used in this study (≤2 vol%), significant segregation or clustering was not observed, consistent with previous studies [36,37]. The fresh HDAAC mixture was cast into molds, vibrated, and leveled. Specimens were covered with plastic film and cured at ambient laboratory conditions for 24 h before demolding. After demolding, specimens were immersed in water for 28 days for curing. Finally, specimens were air-dried in a shaded area before testing.

**Figure 2:**
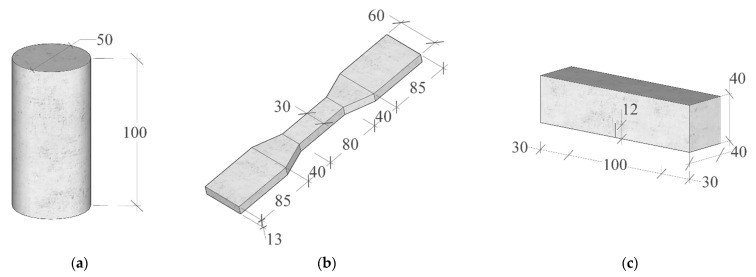
Specimen dimensions (unit: mm): (**a**) cylinder specimen for compressive tests; (**b**) dumbbell-shaped specimen for tensile tests; (**c**) pre-notched specimen for fracture tests.

Figure 2 shows the specimen dimensions used in this study. According to ASTM C469 [38], cylindrical specimens (50 mm in diameter and 100 mm in height; Figure 2a) were used to assess the axial compressive properties of hybrid PP/PE-HDAAC. To evaluate tensile behavior, dumbbell-shaped specimens (Figure 2b) were prepared in line with JSCE guidelines [39]. Notably, this study primarily investigated the fracture performance of hybrid PP/PE-HDAAC thermo-mechanical coupling. For this purpose, notched beam specimens (Figure 2c) were tested following procedures from prior research and established standards [40]. After curing and air-drying under laboratory conditions, the specimens were notched along the center of their longitudinal axis. Each notch had a height of 16 mm (tolerance within ± 0.5 mm) and an opening width of 1 mm. For clear illustration, the numbering of specimens for fracture tests is presented in Table 4. Each test group contained 3 specimens, resulting in a total of 45 specimens.

### 2.3. Testing Setup

#### 2.3.1. Axial Compressive Test

**Figure 3:**
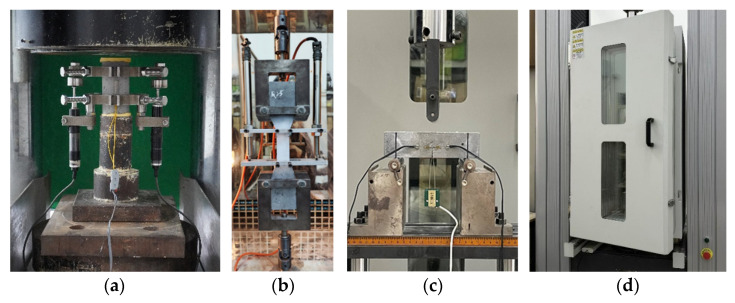
Test setup: (**a**) axial compressive test; (**b**) axial tensile test; (**c**) fracture test; (**d**) temperature chamber.

To evaluate material behavior under axial compression, a 500-ton capacity compression testing machine was used (Figure 3a). Before testing, the top and bottom loading surfaces of each specimen were cleaned and leveled with high-strength gypsum to ensure an even stress distribution. The lateral surfaces were also polished to allow the proper attachment of strain gauges. Axial strain was measured using strain gauges symmetrically bonded to the specimen sides. In addition, two linear variable differential transformers (LVDTs), with a 50 mm gauge length, were installed symmetrically to capture axial deformation. The test was conducted under displacement control at a constant rate of 0.2 mm/min. Throughout the test, axial load, strain, and deformation were recorded in real time using a high-precision data acquisition system, with a sampling rate of 1 Hz.

#### 2.3.2. Axial Tensile Test

The axial tensile test was performed using a microcomputer-controlled electronic universal testing machine (Figure 3b). A displacement-controlled mode was applied throughout the test, with a constant loading rate of 0.5 mm/min. The testing force was continuously recorded by built-in sensors. Meanwhile, two displacement sensors monitored the deformation of the specimen’s central 80 mm section. Data were captured and stored using a high-performance static data acquisition system.

#### 2.3.3. Fracture Test

The fracture performance of the HDAAC was evaluated through three-point bending tests on pre-notched beam specimens (Figure 3c). To begin with, as shown in Figure 3d, the temperature chamber was operated at a heating or cooling rate of ±2.5 °C/min until reaching the target temperature, which was then maintained for 90 min to ensure the uniform heating or cooling of the specimen. Afterwards, testing was conducted on a microcomputer-controlled universal testing machine under displacement control at a loading rate of 0.1 mm/min.

Moreover, specimens were placed on sliding supports to reduce frictional interference and its influence on fracture behavior [41]. Load data were recorded by the machine’s built-in pressure sensor, while mid-span deflection was measured with its integrated displacement sensor. To monitor *CMOD*, a clip-on extensometer was mounted at the notch using two copper sheets bonded with low- and high-temperature-resistant adhesive. Additionally, two strain gauges were horizontally attached 5 mm from either side of the pre-crack tip on one face of the specimen to capture the initial cracking stress, as shown in Figure 3c. Both strain gauge and *CMOD* data were collected using a TDS-540 data acquisition system.

### 2.4. Two-Way Analysis of Variance

**Figure 4:**
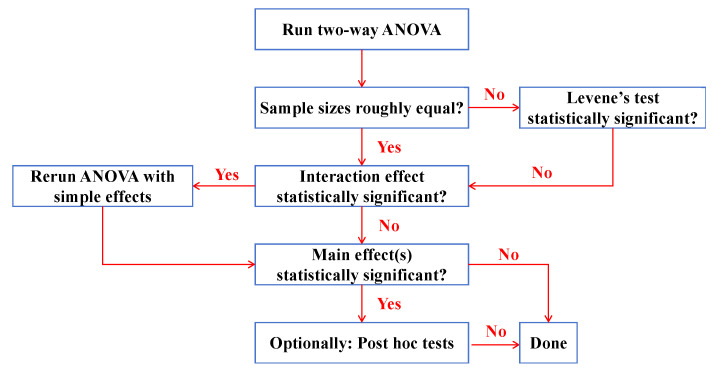
Flowchart of analysis of variance (ANOVA).

Figure 4 illustrates the flowchart of the analysis of variance (ANOVA) performed using IBM SPSS Statistics 27 in this study. A two-way ANOVA was conducted to evaluate the effects of fiber hybrid ratio and coupling temperature on fracture properties of HDAAC in this study. All individual specimen data were included to capture within-group variability. The analysis examined the main effects and the interaction between the two factors mentioned above. Afterwards, post hoc tests were conducted for factor A (fiber replacement ratio: 0%, 25%, and 50%) and factor B (coupled temperature: 0 °C, 30 °C, 70 °C, 100 °C, and 150 °C) using the Bonferroni test, with *p* < 0.05 as the significance threshold (“*p* < 0.001” considered highly significant). Letter groupings were then assigned to each factor level: identical letters indicate no significant difference (*p* ≥ 0.05), while different letters indicate a significant difference (*p* < 0.05). Overlapping letters (e.g., “ab,” “bc”) denote no significant difference with multiple groups, although some pairwise differences may still exist. The letters for factor A and factor B are presented sequentially after each mean ± SD in the format (a, b), corresponding to the main effects of fiber replacement ratio and temperature, respectively. It should be noted the valid samples in this study are fixed at 3, and the sample sizes are roughly equal. The results will be shown in Section 3.3.

## 3. Results

### 3.1. Basic Mechanical Properties of Hybrid PP/PE-HDAAC

Table 5 presents the compressive strength and elastic modulus of hybrid PP/PE-HDAAC. As shown in Table 5, the compressive strength of hybrid PP/PE-HDAAC ranged from 82.4 MPa to 100.5 MPa, and the elastic modulus ranged from 18.1 GPa to 18.7 GPa. Replacing PE fiber with PP fiber led to a decrease in compressive strength, while the elastic modulus showed little change.

**Figure 5:**
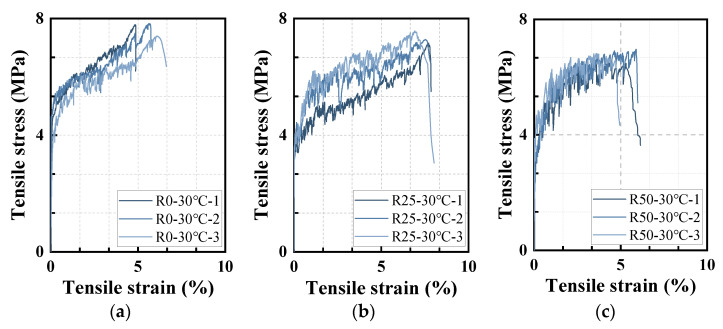
Stress–strain curves of hybrid PP/PE-HDAAC under tension: (**a**) R0-30 °C; (**b**) R25-30 °C; (**c**) R50-30 °C.

Figure 5 presents the tensile stress–strain curves of HDAAC, while Table 6 summarizes the corresponding tensile performance parameters. As expected, the hybrid PP/PE-HDAAC exhibited multiple cracking and strain-hardening behaviors, with noticeable stress fluctuations throughout the loading process. According to Table 6, replacing PP fibers with PE fibers resulted in a reduction in the initial cracking strength—from 4.4 MPa to 2.7 MPa. Although the tensile strength remained relatively stable, the ultimate tensile strain reached its maximum at a 25% PP fiber replacement ratio (R25-30 °C).

### 3.2. Fracture Properties

#### 3.2.1. Failure Mode

**Figure 6:**
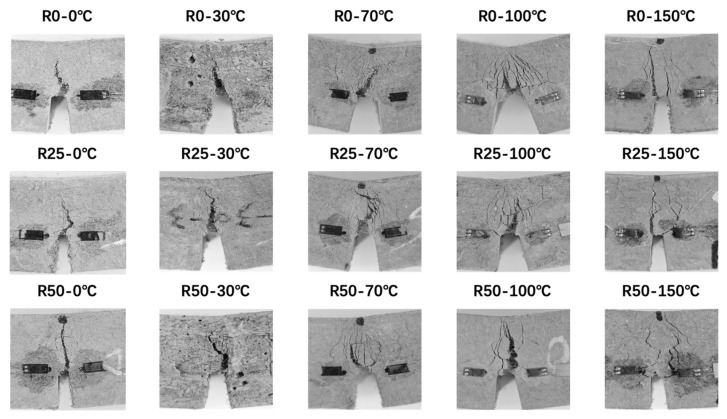
Failure modes of notched specimens.

Figure 6 illustrates the failure modes of notched specimens, with the images being processed for better understanding. As shown in Figure 6, all specimens developed a prominent main crack within the fracture process zone under the combined effects of thermal and mechanical loading, accompanied by numerous fine surrounding cracks. Initially, cracking began at the prefabricated notch tip at the onset of loading, though the crack was barely visible. As loading gradually progressed, the main crack slowly propagated along the notch direction, while a growing number of microcracks emerged around the crack tip. Fiber pull-out and rupture occurred during the whole process. In addition, the surface of the main crack was irregular and curved, increasing the fracture surface area and reducing stress concentration at the crack tip [42]. Eventually, as the fiber-bridging capacity weakened, both the main crack and adjacent microcracks expanded rapidly, resulting in complete specimen failure. It should be noted that crack patterns varied noticeably with temperature. At 0 °C, crack propagation was restrained, leading to narrower main and secondary cracks. At 70 °C and 100 °C, the number of microcracks increased substantially, indicating enhanced ductility. At 150 °C, despite the thermal degradation of the fibers—including melting damage and reductions in elastic modulus and tensile strength—a residual bridging effect persisted. As a result, multiple cracks around the main crack were still evident.

#### 3.2.2. *P-δ* Curves and *P-CMOD* Curves

**Figure 7:**
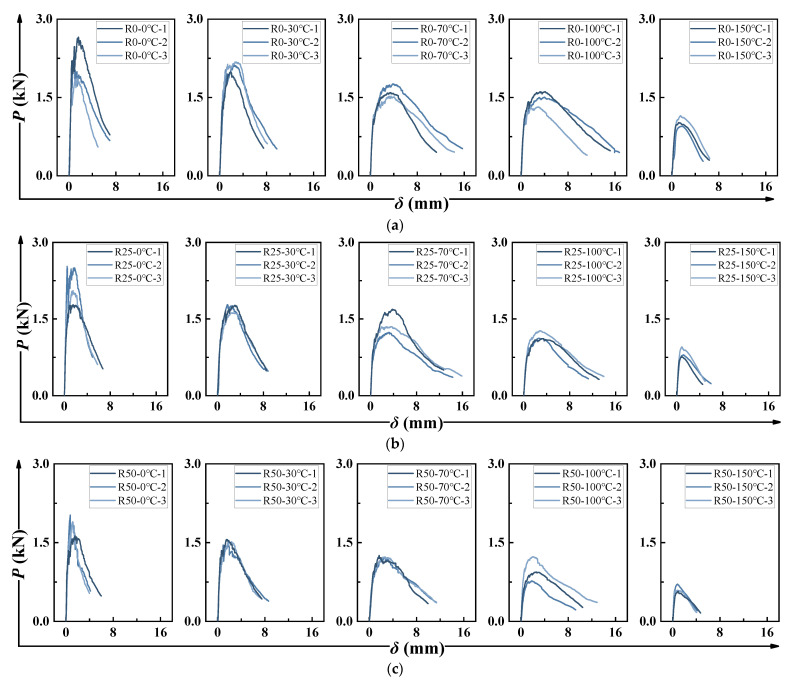
*P-δ* curves: (**a**) R0 group; (**b**) R25 group; (**c**) R50 group.

**Figure 8:**
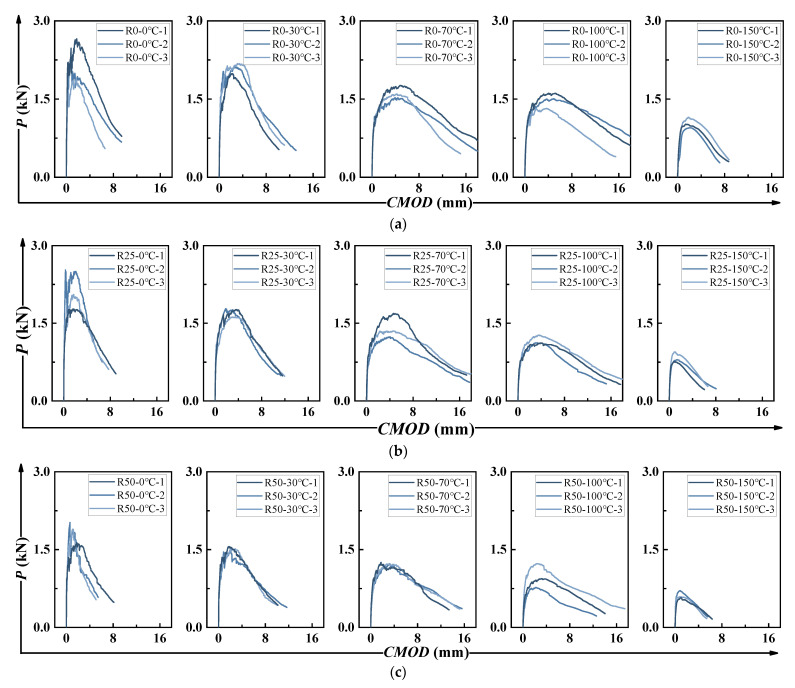
*P-CMOD* curves: (**a**) R0 group; (**b**) R25 group; (**c**) R50 group.

Figure 7 and Figure 8 show the *P-δ* and *P-CMOD* curves, respectively. Both curves displayed similar trends. For all HDAAC notched specimens, strain-hardening behavior was evident after the initial cracking, indicating favorable ductility. The strain-hardening phenomenon became more pronounced as temperature increased—up to 100 °C. However, at 150 °C, the material’s ductility declined significantly. For instance, fewer and wider cracks were observed when it came to R50-150 °C. It indicated the loss of steady-state multiple cracking behavior and premature crack localization, which explained the reduction in ductility. A similar degradation trend was seen with increasing PP fiber content. Comparable behavior has been reported in previous studies [30]. It has been shown that the fracture process of pseudo strain-hardening concrete materials can be described in six characteristic stages, which is appliable to this study as well. A distinguishing feature of the HDAAC fracture process was the multiple crack development stage, characterized by the simultaneous formation of new cracks around the crack tip and the propagation of existing ones. During this stage, the applied load remained relatively stable.

#### 3.2.3. Mode I Fracture Energy of Hybrid PP/PE-HDAAC

Mode I fracture energy *G*_F_ specifically refers to the strain energy release rate at the crack tip. Since mode I fracture is considered the most critical fracture mode, this section examined how the temperatures and PP fiber replacement ratios influenced the *G*_F_ of HDAAC in this study. *G*_F_ was calculated following Equations (1) and (2) as suggested in JCI-S-001-2003 [40].

(1) $$G_{F}=\frac{0.75W_{0}+W_{1}}{A_{lig}}$$

(2) $$W_{1}=0.75\left(\frac{S}{L}m_{1}+2m_{2}\right)g\cdot {CMOD}_{0}$$

The fracture energy calculation incorporates the following parameters:

*W*_0_: The area under the *P-CMOD* curve, representing the total mechanical work input.

*W*_1_: The work done by the weight of the specimen and its supporting fixture.

*A*_lig_: The cross-sectional area of the uncracked ligament.

*S*/*L*: The ratio of the test span length to the specimen length.

*m*_1_: The mass of the specimen.

*m*_2_: The mass of fixture components not rigidly clamped to the testing frame (typically assumed negligible, i.e., *m*_2_ = 0)

g: Gravitational acceleration, taken as 9.81 m/s^2^.

Conventionally, *CMOD*_0_ is defined as the *CMOD* value at which the load drops to zero. However, to account for the enhanced deformation capacity of HDAAC, this study considered *CMOD*_0_ as the *CMOD* corresponding to 30% of the peak load during the unloading phase.

**Figure 9:**
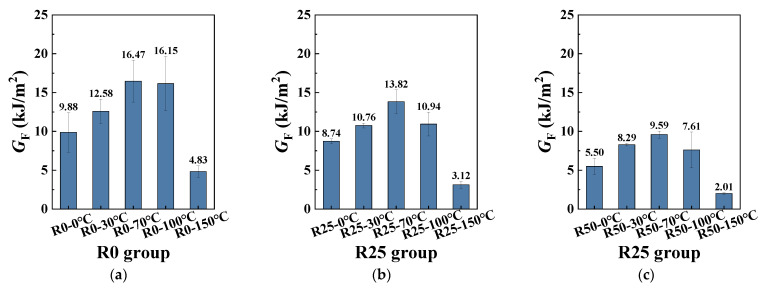
Variation in mode I fracture energy *G*_F_: (**a**) R0-30 °C; (**b**) R25-30 °C; (**c**) R50-30 °C.

Table 7 and Figure 9 illustrate the results and variations in the mode I fracture energy *G*_F_ of hybrid PP/PE-HDAAC. In summary, the *G*_F_ maintained a high level at any PP fiber replacement ratio while declining to a relatively low level at 150 °C. In addition, increasing the coupled temperature led to an obvious influence of *G*_F_. For instance, as shown in Table 7 and Figure 9, the *G*_F_ of R0-30 °C, R25-30 °C, and R50-30 °C was 12.58 kJ/m^2^, 10.76 kJ/m^2^, and 8.29 kJ/m^2^, respectively. It can be observed that the *G*_F_ of hybrid PP/PE-HDAAC gradually declined as the PP fiber replacement ratio increased. In addition, when the coupled temperature increased from 0 °C to 70 °C, the *G*_F_ of R0-70 °C increased by 66.7% compared with that of R0-0 °C. However, when it came to 150 °C, the *G*_F_ showed a sharp decrease for each group. For instance, the *G*_F_ of R0-150 °C fell to 4.83 kJ/m^2^, which was only one third of the *G*_F_ of R0-100 °C.

#### 3.2.4. Fracture Energy Based on J-Integral Method

As previously discussed, the classical double-K fracture model is unsuitable for HDAAC-type pseudo strain-hardening concretes, as its fundamental assumptions are not met [22,43]. Consequently, it cannot be reliably used to evaluate the fracture performance of HDAAC in this study. In accordance with the approach proposed in the study of Marshall and Cox [44] and Su et al. [45], this study adopted the J-integral method—originating from post-yield fracture mechanics—to characterize the fracture process of hybrid PP/PE-HDAAC under thermo-mechanical coupling from an energy-based perspective. In the present study, multiple microcracks were observed to develop around the crack tip of notched specimens. However, failure was ultimately governed by the propagation of a dominant crack. This fracture behavior justifies the use of the J-integral method for characterizing the energy evolution during cracking. The J-integral approach allows for the evaluation of both initiation fracture energy, representing the energy released during the formation of the initial crack (*J*_IC_), and failure fracture energy, corresponding to the energy released during unstable fracture (*J*_IF_). The total fracture energy of the composite (*J*_C_) represents the overall energy dissipation during crack propagation and consists of two primary components: the energy dissipated by the alkali-activated matrix outside the crack surface during crack extension (*J*_m_) and the energy consumed by fiber-bridging mechanisms within the crack surface (*J*_b_). The specific calculation formulas are given as follows:

(3) $$J_{C}=\frac{2S_{C}}{B\left(W-a_{0}\right)}$$

(4) $$J_{IC}=\frac{2S_{IC}}{B\left(W-a_{0}\right)}$$

(5) $$J_{IF}=\frac{2S_{IF}}{B\left(W-a_{0}\right)}$$

(6) $$J_{b}=U/A_{lig}$$

(7) $$J_{m}=J_{C}-J_{b}$$

The above formulas include some basic parameters as follows: *S*_c_ denotes the integral of the *P-δ* curve. *B* and *W* represent the specimen width and height, respectively. a_0_ indicates the pre-cut notch depth. *S*_IC_ corresponds to the area under the *P-δ* curve from the origin to the initial crack point (*P*_ini_), while *S*_IF_ is derived from the integral up to the failure load. *U* is quantified by the area under the *P-CMOD* response, with *A*_lig_ being the net cross-sectional area of the ligament.

**Figure 10:**
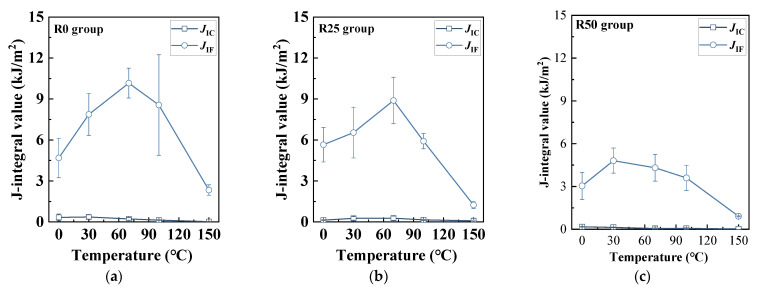
Variation of *J*_IC_, *J*_IF_: (**a**) R0 group; (**b**) R25 group; (**c**) R50 group.

**Figure 11:**
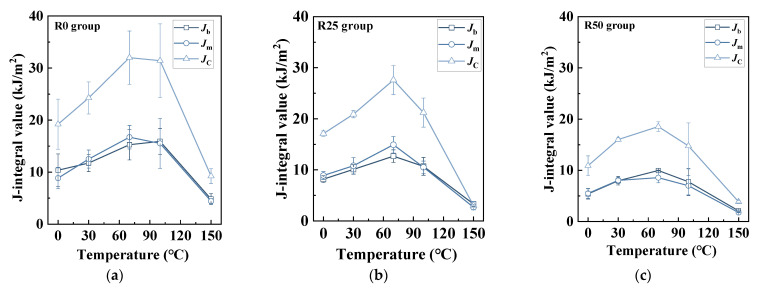
Variation of *J*_b_, *J*_m_, and *J*_c_: (**a**) R0 group; (**b**) R25 group; (**c**) R50 group.

Table 8 and Figure 10 and Figure 11 display the results of fracture energy based on the J-integral method. To begin with, it can be found that the *J*_IC_ was at a magnitude of 0.1 kJ/m^2^, similar to the study of Ding et al. [46]. It also showed inapparent patterns when PP fiber replacement ratio and coupled temperature changed. Notably, the *J*_IF_ soared compared with *J*_IC_, implying that a great deal of energy was necessary to come up to the unstable fracture stage for HDAAC. Specifically, the highest *J*_IF_ value was 7.87 kJ/m^2^ (R0-30 °C), yet it declined when the PP fiber replacement ratio increased. Nonetheless, at a replacement ratio of 50%, the *J*_IF_ value was 4.81 kJ/m^2^, which was still a few dozen times that of *J*_IC_. In addition, the *J*_IF_ generally peaked at 70 °C, as shown in Figure 10. For instance, the highest *J*_IF_ value was 10.16 kJ/m^2^ for R0-70 °C.

Similarly, an increased coupled temperature led to a more obvious ductile fracture mode, specifically resulting in an increase in *J*_m_ and *J*_b_. As mentioned above, *J*_m_ represents the consumed energy of matrix cracking, while *J*_b_ represents the consumed energy related with fiber pull-out and fracture. From Figure 11, both *J*_m_ and *J*_b_ increased as coupled temperature ascended and peaked at 70 °C. For instance, the *J*_m_ of R0-70 °C was approximately two times that of R0-0 °C. However, the *J*_m_ of R0-150 °C declined to nearly 25% of that of R0-70 °C. Similar patterns were observed in terms of *J*_b_ as well. For another, *J*_m_ and *J*_b_ showed a decreasing trend with an increasing PP fiber replacement ratio. Still, the descent of *J*_m_ and *J*_b_ was moderate. For example, the *J*_m_ and *J*_b_ of R25-30 °C decreased by 13.6% and 14.1%, respectively. However, the *J*_m_ and *J*_b_ of R50-30 °C decreased by 35.7% and 32.2%, respectively. Notably, it can be observed that the values of *J*_m_ and *J*_b_ was nearly kept at the same level (*J*_m_/*J*_c_ = 0.4–0.5), which was distinguished from conventional concrete and FRC.

### 3.3. Significance Analysis

Since the relevant data are already presented in previous tables as means and standard deviations, no additional descriptive statistics table is provided. Table 9 summarizes the core results of the two-way analysis of variance, showing the main effects of factor A (fiber hybrid ratio), factor B (coupled temperature), and their interaction on the fracture performance indicators (*G*_F_, *J*_IC_, *J*_IF_, *J*_c_, *J*_b_, and *J*_m_) examined in this study. As shown in Table 9, the interaction between fiber ratio and temperature was not significant for any indicators (*p* > 0.2; see Table 9), indicating that the effect of fiber ratio is largely independent of temperature. Partial eta squared (η^2^) values for the interaction were also relatively small (η^2^ = 0.178–0.285), confirming that the contribution of the interaction to the total variance is minimal. In contrast, both the fiber hybrid ratio and the temperature exhibited significant main effects (*p* < 0.05), with medium to large effect sizes. Specifically, the η^2^ for fiber ratio ranged from 0.217 to 0.640, indicating that fiber content has a substantial impact on most fracture properties. Temperature exhibited the largest effects across all indicators (η^2^ = 0.356–0.818), indicating that thermal exposure is the dominant factor influencing fracture performance. Overall, the results show that fiber content and temperature independently affect fracture properties, with temperature generally having the strongest impact, while their interaction is minimal. These findings underscore the importance of considering both fiber hybrid ratio and thermal conditions separately when optimizing HDAAC’s performance.

Furthermore, post hoc tests were conducted and the results of pairwise comparisons are presented in Table 10 and Table 11. The fiber replacement ratio (factor A) significantly affected most fracture performance indicators, with pairwise comparisons generally following the trend R0 > R25 > R50 (*p* < 0.05). In contrast, *J*_IC_ showed weaker sensitivity. The differences between R0 and R25 and between R25 and R50 were not significant, while only R0 and R50 differed significantly (*p* = 0.024). Coupled temperature (factor B) had a stronger influence on toughness. Apart from *J*_IC_, all indicators decreased significantly with rising coupled temperature, with values at 150 °C markedly lower than those at other levels (*p* < 0.001). For *J*_IC_, no significant differences were observed within 0 °C to 100 °C, but a sharp decrease occurred at 150 °C (*p* = 0.008), indicating that *J*_IC_ is primarily sensitive to high-temperature exposure.

Overall, both the fiber replacement ratio and the coupled temperature exerted significant main effects on the fracture performance indicators, with temperature being the dominant factor. The general trend shows that increasing fiber replacement ratio or service temperature reduces ductility, with high-temperature exposure as the critical driver of degradation.

## 4. Discussion

### 4.1. Basic Mechanical Properties of Hybrid PP/PE-HDAAC Under Ambient Temperature

From the test results, it can be found that fiber hybridization showed little influence on the compressive properties since both the PP and PE fiber were flexible fiber. Conversely, the effect of fiber hybridization on tensile performance was more apparent. Given the lower mechanical properties of PP fibers than those of PE fibers, the effect of fiber hybridization refers to the fiber-bridging capacity of added fibers. As a result, a decrease in initial cracking strength and tensile strength can be observed in this study. As for the ultimate tensile strain, the peak value (7.4%) was found at a fiber replacement ratio of 25% while declining to 5.3% when the fiber replacement ratio grew to 50%. This phenomenon was primarily governed by the HDAAC’s crack control capacity which decreased when the PP fiber replacement ratio increased. Specifically, it was manifested by a decrease in crack density and an increase in crack width, which remained key variables in regulating the macroscopic deformation. Consequently, with increasing PP fiber content, the ultimate tensile strain exhibited the aforementioned non-monotonic variation.

### 4.2. Ductile Fracture Phenomenon of Hybrid PP/PE-HDAAC

The fracture behavior of hybrid PP/PE-HDAAC clearly differed from the brittle response of conventional mortar and concrete, exhibiting a ductile failure mode similar to that reported for ECC [42,43,47,48]. Under ambient conditions (30 °C in this study), the total fracture energy *J*_C_ reached approximately 30 kJ/m^2^ at most, whereas conventional concrete or FRC typically exhibited fracture energy values of only several kJ/m^2^ at ambient temperature. It was primarily attributed to the formation and propagation of microcracks within the fracture process zone, along with fiber pull-out and rupture. Final fracture in HDAAC occurred only when the stress concentration at the crack tip surpasses the fiber-bridging capacity, as described in previous studies [8,9,49,50]. Such ductile fracture phenomenon led to the high values of *J*_m_ and *J*_b_, which were quite different from conventional concrete.

A notable feature was the large gap between *J*_IC_ and *J*_IF_. *J*_IC_ values were relatively small—on the order of 0.1 kJ/m^2^—comparable to those of ordinary cement mortar, as reported in the literature [47]. This low initiation energy was primarily due to the low sand-to-binder ratio. However, as shown in Table 8, the *J*_IF_ were several to dozens of times higher than the corresponding *J*_IC_, showcasing that HDAAC was capable of absorbing a substantial amount of energy after crack initiation before reaching unstable fracture. Such a marked delay in fracture failure underscored the superior fracture resistance and energy absorption capacity of the hybrid PP/PE-HDAAC.

Furthermore, both the *J*_m_ and *J*_b_ of hybrid PP/PE-HDAAC peaked at 70 °C and dropped to a minimal value at 150 °C. In the study of Chen, Liu, Li, Lin and Guo [37], it has been shown that rising temperatures led to a gradual decrease in the Ca/Si ratio of the alkali-activated matrix, indicating enhanced FA hydration and increased silicon dissolution. This reduction in the Ca/Si ratio weakened the matrix fracture toughness, making it more prone to cracking and thereby contributing to a higher *J*_m_. The trend in *J*_b_ closely parallelled that of *J*_m_, as a more crack-prone matrix allowed fibers to more effectively engage in crack bridging. While fiber hybridization generally led to a reduction in both *J*_m_ and *J*_b_, a 25% replacement ratio of PP fibers maintained relatively high values for both parameters.

### 4.3. Fracture Characteristics of HDAAC Under Thermo-Mechanical Loadings

Overall, the effect of temperature on HDAAC in the thermo-mechanical coupling test was similar to that observed in the residual fracture performance test. The key difference between them lay in the timing of fiber exposure to elevated temperatures. In this study, fibers endured both thermal and mechanical loads simultaneously. As a result, HDAAC’s deformation also included that of fibers, which became significantly more pronounced at elevated temperatures. This was primarily due to the melting points of PP and PE fibers (114–152 °C for PE fibers and lower for PP fibers [37]). As the temperature approached the critical temperature, the fibers experienced substantial thermal softening, accompanied by marked reductions in strength and elastic modulus. As a result, a significant decline in *J*_b_ was observed once the temperature exceeded a critical threshold (the melting point of fibers)—for example, 2.10 kJ/m^2^ for the R50-150 °C. Consequently, HDAAC was no longer capable of exhibiting a ductile failure mode, and failure occurred immediately upon crack initiation.

**Figure 12:**
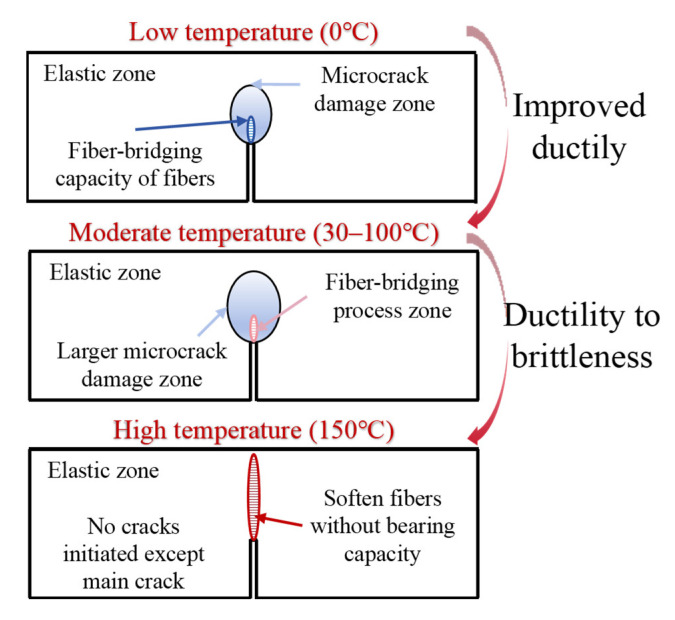
Schematic illustrations of fracture mechanism of hybrid PP/PE-HDAAC.

In order to fully illuminate the fracture mechanism of hybrid PP/PE-HDAAC under thermal loading, Figure 12 shows the fracture characteristics of HDAAC under different conditions. It can be seen that the fracture ductility of HDAAC improves as coupled temperature increases. Nevertheless, when it exceeds the fiber melting temperature of fibers (150 °C for instance), a transition from ductility to brittleness was observed. As shown in Figure 12, the matrix-cracking phenomenon was the most apparent, yet it entirely disappears when the temperature rises to 150 °C.

In summary, the fracture behavior of HDAAC largely depends on the mechanical properties of the polymer fibers under high-temperature service conditions. The observed reduction in the fracture performance of hybrid PE/PP-HDAAC is primarily attributed to the strength loss and thermal softening of the polymer fibers. Under thermo-mechanical loading, polymer fibers may also undergo thermal softening, viscoelastic deformation, creep, and interface changes, all of which reduce the fiber-bridging capacity and further influence the fracture performance. Consequently, the quantitative results of this study are fiber-dependent. The main conclusions are applicable to HDAAC incorporating polymer fibers that exhibit thermal softening and strength loss. However, for other types of heat-resistant polymer fibers (e.g., aramid fibers), these quantitative findings cannot be directly applied and require separate experimental validation. From a practical engineering perspective, a PP fiber replacement ratio of 25% can optimize tensile strain under ambient conditions. In addition, a replacement ratio of 50% can maintain the tensile and fracture performance while further lowering the cost. From the perspective of fracture performance, HDAAC can be applied when the temperature ranges from 0 °C to 100 °C. When it exceeds 100 °C, 150 °C for instance, HDAAC is no longer applicable due to the entire loss of the excellent tensile and fracture performance.

### 4.4. Future Research

(1) Cost control and fiber efficiency. Reducing the cost of HDAAC-type composites remains crucial. Advanced fiber hybridization or ultra-low fiber designs can control the material cost, but their effects on performance must be evaluated. For example, Wang, Ma, Ding, Yu and Yu [12] developed an HDAAC with only 0.2% fiber by volume that still achieved high ductility; the low fracture toughness matrix enabled its performance and the embodied cost was only 30% of a conventional high-fiber ECC. Future work should similarly explore hybrid fiber systems and tailor the matrix toughness so as to minimize the fiber content without sacrificing strength or toughness.
(2) The exploration of fiber surface treatments. Optimizing fiber surface treatment is another promising direction. Treatments (e.g., alkali washes, silane coupling agents, or nanoparticle coatings) can remove impurities and enhance fiber–matrix bonding, which can increase composites’ ductility. Future studies should systematically vary and optimize such treatments (and even develop novel coatings) for the fibers of HDAAC to strengthen the fiber–matrix interface and improve the mechanical properties.
(3) Improve high-temperature resistance. Improving thermal–mechanical performance requires both heat-resistant matrices and fibers. Binders like low-CO_2_ cements (e.g., LC3 or other blended cements) or alkali-activated materials can be combined with heat-resistant fibers. Promising fiber alternatives include aramid and PBO fibers (as already noted) as well as basalt, carbon, or ceramic fibers [51]. Research should evaluate these and other emerging fibers (and matrix modifications) in HDAAC to extend its service temperature range and fire resistance.
(4) Modeling and simulation approaches. Computational modeling can greatly aid design optimization. Multi-scale numerical methods (e.g., the finite element analysis of the composite specimen or micromechanical models of fiber–matrix cracking) can predict how changes in fiber content, orientation, or interface properties affect behavior. Future work should develop models for HDAAC under combined thermal and mechanical loads, allowing the prediction of ductility, crack patterns, and failure mechanisms without relying solely on experiments.
(5) Durability under cyclic and long-term loading. The long-term reliability of HDAAC under service conditions is still not well understood. Future research should address its fatigue behavior under cyclic and long-term loading, with a particular emphasis on crack evolution, fiber–matrix interface, and the degradation of mechanical properties. Sustained loading effects such as creep and shrinkage-induced cracking also deserve attention, as they directly influence service life and structural safety.

## 5. Conclusions

In this study, the fracture performance of hybrid PP/PE-HDAAC was systematically investigated under varying PP fiber replacement ratios (0%, 25%, and 50%) and coupled thermal–mechanical loading conditions (0 °C, 30 °C, 70 °C, 100 °C, and 150 °C). This research primarily aimed to illuminate the effects and mechanisms of thermo-mechanical coupling on the fracture behavior. The key aspects analyzed included the crack evolution process and mode I fracture energy *G*_F_. Furthermore, the fracture process was evaluated from an energy release perspective using the J-integral method. The main conclusions are summarized as follows:

(1) Overall, except at 150 °C, hybrid PP/PE-HDAAC exhibited a ductile fracture mode under all experimental conditions up to 100 °C. Although the failure mode of the notched beam specimens was still characterized by a primary crack propagating along the notch direction, numerous microcracks developed around the crack tip during the multiple cracking stage of the *P-CMOD* curve. During this stage, the applied load remained relatively stable.
(2) Under varying thermo-mechanical coupling temperatures, the fracture energy of hybrid PP/PE-HDAAC generally remained high, reaching up to 16.47 kJ/m^2^, with a sharp decline only at 150 °C, where it dropped to 2.01 kJ/m^2^. This reduction was attributed to fiber melting at elevated temperatures, which severely compromised the fiber-bridging effect and resulted in a transition from ductile to brittle failure.
(3) The *J*_IC_ remained consistently around 0.1 kJ/m^2^, showing minimal sensitivity to changes in temperature and fiber replacement ratio. In contrast, the *J*_IF_ was significantly higher, reflecting the HDAAC’s excellent energy dissipation capacity after crack initiation. This enhanced fracture performance was mainly due to multiple cracking phenomenon around the crack tip and stress redistribution, both of which delayed final failure. *J*_IF_ reached its maximum at 70 °C (e.g., 10.16 kJ/m^2^ for the R0 group), but declined sharply at 150 °C, indicating that excessively high temperatures reduced the ability to sustain ductile fracture behavior.
(4) Both *J*_m_ and *J*_b_ increased with temperature, peaking at 70 °C, followed by a substantial drop at 150 °C. The ratio *J*_m_/*J*_c_ remained relatively stable between 0.4 and 0.5, suggesting that matrix cracking contributed to approximately half of the total fracture energy. This kind of fracture behavior was distinct from that of conventional concrete and typical FRC. Increasing the PP fiber replacement ratio led to a reduction in both *J*_m_ and *J*_b_, with the most pronounced decrease observed at the 50% PP fiber replacement ratio. Under thermo-mechanical coupling, fibers were subjected to simultaneous thermal and mechanical loads. Elevated coupled temperatures caused fiber softening or melting, severely diminishing the fiber-bridging capacity. At 150 °C, fibers were no longer able to support the stable growth of matrix cracking, resulting in a transition to brittle failure.

## Figures and Tables

**Table 1:** Chemical composition and physical properties of FA and GGBS (unit: wt%).

	CaO	SiO_2_	Al_2_O_3_	SO_3_	Fe_2_O_3_	MgO	TiO_2_	Other	LOI (%)
GGBS	34.0	34.5	17.7	1.64	1.03	6.01	/	5.12	0.840
FA	4.01	54.8	31.2	2.20	4.16	1.01	1.13	2.37	4.60

Note: LOI denotes loss on ignition.

**Table 2:** Physical and mechanical properties of PE and PP fiber.

Fiber Type	Length (mm)	Diameter (um)	Strength (MPa)	Density (g/cm^3^)	Elastic Modulus (GPa)
PP	12.0	100.0	400	0.910	13.5
PE	12.0	20.0	3000	0.970	120

**Table 3:** Mix proportions of hybrid PP/PE-HDAAC.

Group	FA ^1^	GGBS ^2^	QS ^3^	AAS ^4^	Water	BaCl_2_	Volume Dosage (Vol. %)	
							PE	PP
R0	0.70	0.30	0.20	0.40	0.06	0.01	2.0	0
R25	0.70	0.30	0.20	0.40	0.06	0.01	1.5	0.5
R50	0.70	0.30	0.20	0.40	0.06	0.01	1.0	1.0

^1^ fly ash; ^2^ ground granulated blast furnace slag; ^3^ quartz sand; ^4^ alkali-activated solution.

**Table 4:** Grouping of specimens for different tests.

Group	Specimen IDs	Temperature (°C)	Number	Compressive Test	Tensile Test	Fracture Test
R0	R0-0 °C *	0	3	×	×	√
	R0-30 °C	30	3	√	√	√
	R0-70 °C	70	3	×	×	√
	R0-100 °C	100	3	×	×	√
	R0-150 °C	150	3	×	×	√
R25	R25-0 °C	0	3	×	×	√
	R25-30 °C	30	3	√	√	√
	R25-70 °C	70	3	×	×	√
	R25-100 °C	100	3	×	×	√
	R25-150 °C	150	3	×	×	√
R50	R50-0 °C	0	3	×	×	√
	R50-30 °C	30	3	√	√	√
	R50-70 °C	70	3	×	×	√
	R50-100 °C	100	3	×	×	√
	R50-150 °C	150	3	×	×	√

* The fiber dosage is the volume ratio of the external dosage. Ra-b °C represents the PP mixing rate as a/% and the temperature is b/°C.

**Table 5:** Compressive properties of hybrid PP/PE-HDAAC.

Specimen IDs	Compressive Strength (MPa)	Elastic Modulus (GPa)
R0-30 °C	100.5 ± 3.9	18.7 ± 0.6
R25-30 °C	102.8 ± 3.6	19.4 ± 0.0
R50-30 °C	82.4 ± 5.6	18.1 ± 0.5

Note: The values represent the average ± standard deviation.

**Table 6:** Tensile properties of hybrid PP/PE-HDAAC.

Specimen IDs	Initial Cracking Strength (MPa)	Tensile Strength (Mpa)	Ultimate Tensile Strain (%)
R0-30 °C	4.4 (0.3)	7.7 (0.2)	5.5 (0.5)
R25-30 °C	3.5 (0.2)	7.3 (0.2)	7.4 (0.3)
R50-30 °C	2.7 (0.8)	7.7 (0.2)	5.3 (0.5)

Note: The values represent the average ± standard deviation.

**Table 7:** Results of mode I fracture energy *G*_F_.

Mix IDs	*m* (kg)	*CMOD*_0_ (mm)	*W*_0_ (kN·mm)	*W*_1_ (kN·mm)	*G*_F_ (kJ/m^2^)
R0-0 °C	0.485 ± 0.010	8.46 ± 1.34	12.62 ± 3.25	0.023 ± 0.004	9.88 ± 2.54 a, a
R0-30 °C	0.504 ± 0.001	11.46 ± 1.21	16.06 ± 2.00	0.032 ± 0.004	12.58 ± 1.56 a, a
R0-70 °C	0.491 ± 0.004	18.63 ± 2.61	21.01 ± 3.43	0.051 ± 0.007	16.47 ± 2.69 a, b
R0-100 °C	0.498 ± 0.006	19.49 ± 2.97	20.61 ± 4.47	0.054 ± 0.008	16.15 ± 3.50 a, c
R0-150 °C	0.501 ± 0.012	8.21 ± 0.73	6.15 ± 0.96	0.023 ± 0.002	4.83 ± 0.76 a, d
R25-0 °C	0.489 ± 0.012	7.78 ± 0.96	11.16 ± 0.39	0.021 ± 0.003	8.74 ± 0.31 b, a
R25-30 °C	0.487 ± 0.005	11.61 ± 0.27	13.73 ± 0.41	0.031 ± 0.001	10.76 ± 0.32 b, a
R25-70 °C	0.492 ± 0.004	18.23 ± 1.11	17.62 ± 1.95	0.050 ± 0.003	13.82 ± 1.52 b, b
R25-100 °C	0.491 ± 0.012	17.34 ± 1.63	13.95 ± 1.97	0.047 ± 0.004	10.94 ± 1.54 b, c
R25-150 °C	0.492 ± 0.006	6.91 ± 0.85	3.97 ± 0.58	0.019 ± 0.002	3.12 ± 0.45 b, d
R50-0 °C	0.472 ± 0.006	6.22 ± 1.36	7.02 ± 1.36	0.016 ± 0.004	5.50 ± 1.07 c, a
R50-30 °C	0.471 ± 0.002	10.65 ± 0.73	10.57 ± 0.22	0.028 ± 0.002	8.29 ± 0.17 c, a
R50-70 °C	0.481 ± 0.010	14.75 ± 0.96	12.2 ± 0.59	0.039 ± 0.003	9.59 ± 0.46 c, b
R50-100 °C	0.498 ± 0.006	14.68 ± 2.03	9.69 ± 2.95	0.040 ± 0.006	7.61 ± 2.31 c, c
R50-150 °C	0.490 ± 0.010	5.78 ± 0.40	2.55 ± 0.15	0.016 ± 0.001	2.01 ± 0.12 c, d

Note: The values represent the average ± standard deviation; for the two letters following each *G*_F_ value, the left denotes the main effect of fiber replacement ratio, and the right denotes the main effect of coupled temperature.

**Table 8:** Results of fracture energy based on J-integral method.

Mix IDs	*J*_IC_ (kJ/m^2^)	*J*_IF_ (kJ/m^2^)	*J*_c_ (kJ/m^2^)	*J*_b_ (kJ/m^2^)	*J*_m_ (kJ/m^2^)	*J*_m_/*J*_c_
R0-0 °C	0.33 ± 0.25 a, a	4.68 ± 1.44 a, a	19.21 ± 4.81 a, c	10.37 ± 3.12 a, b	8.85 ± 2.04 a, b	0.47 ± 0.06
R0-30 °C	0.36 ± 0.16 a, a	7.87 ± 1.54 a, a	24.26 ± 3.09 a, bc	11.75 ± 1.65 a, ab	12.51 ± 1.74 a, ab	0.52 ± 0.03
R0-70 °C	0.22 ± 0.10 a, a	10.16 ± 1.09 a, b	31.99 ± 5.13 a, a	15.27 ± 2.93 a, a	16.72 ± 2.27 a, a	0.53 ± 0.02
R0-100 °C	0.13 ± 0.04 a, a	8.56 ± 3.68 a, a	31.42 ± 7.07 a, ab	15.91 ± 2.49 a, a	15.51 ± 4.78 a, a	0.48 ± 0.06
R0-150 °C	0.02 ± 0.01 a, b	2.33 ± 0.39 a, c	9.24 ± 1.44 a, d	4.82 ± 1.05 a, c	4.42 ± 0.51 a, c	0.48 ± 0.05
R25-0 °C	0.13 ± 0.04 ab, a	5.65 ± 1.26 a, a	17.09 ± 0.55 b, c	8.18 ± 0.66 b, b	8.90 ± 0.76 b, b	0.52 ± 0.04
R25-30 °C	0.26 ± 0.13 ab, a	6.54 ± 1.86 a, a	20.90 ± 0.69 b, bc	10.09 ± 0.92 b, ab	10.81 ± 1.60 b, ab	0.52 ± 0.06
R25-70 °C	0.27 ± 0.16 ab, a	8.89 ± 1.70 a, b	27.59 ± 2.83 b, a	12.68 ± 1.23 b, a	14.92 ± 1.62 b, a	0.54 ± 0.01
R25-100 °C	0.15 ± 0.04 ab, a	5.91 ± 0.56 a, a	21.21 ± 2.85 b, ab	10.69 ± 1.74 b, a	10.52 ± 1.20 b, a	0.50 ± 0.02
R25-150 °C	0.08 ± 0.05 ab, b	1.24 ± 0.25 a, c	3.00 ± 0.85 b, d	3.32 ± 0.47 b, c	2.68 ± 0.42 b, c	0.45 ± 0.02
R50-0 °C	0.17 ± 0.01 b, a	3.04 ± 0.95 b, a	10.91 ± 1.92 c, c	5.40 ± 1.01 c, b	5.50 ± 0.98 c, b	0.51 ± 0.03
R50-30 °C	0.13 ± 0.02 b, a	4.81 ± 0.88 b, a	16.01 ± 0.35 c, bc	7.97 ± 0.83 c, ab	8.04 ± 0.60 c, ab	0.50 ± 0.04
R50-70 °C	0.05 ± 0.03 b, a	4.31 ± 0.94 b, b	18.54 ± 0.94 c, a	9.97 ± 0.07 c, a	8.57 ± 0.99 c, a	0.46 ± 0.03
R50-100 °C	0.06 ± 0.03 b, a	3.60 ± 0.89 b, a	14.77 ± 4.50 c, ab	7.77 ± 2.59 c, a	7.00 ± 1.95 c, a	0.48 ± 0.02
R50-150 °C	0.02 ± 0.00 b, b	0.91 ± 0.03 b, c	3.87 ± 0.23 c, d	2.10 ± 0.17 c, c	1.77 ± 0.05 c, c	0.46 ± 0.01

Note: The values represent the average ± standard deviation; for the two letters following each *G*_F_ value, the left denotes the main effect of fiber replacement ratio, and the right denotes the main effect of coupled temperature.

**Table 9:** Results of two-way ANOVA.

Source	*A* × *B*	*A*	*B*
*G*_F_ (F, *p*, η^2^)	1.125, 0.375, 0.231	26.661, <0.001 ***, 0.640	33.037, <0.001 ***, 0.815
*J*_IC_ (F, *p*, η^2^)	1.007, 0.451, 0.212	4.159, 0.025 *, 0.217	4.145, 0.009 **, 0.356
*J*_IF_ (F, *p*, η^2^)	1.284, 0.289, 0.255	14.493, <0.001 ***, 0.491	16.787, <0.001 ***, 0.691
*J*_c_ (F, *p*, η^2^)	1.214, 0.325, 0.245	26.510, <0.001 ***, 0.639	33.756, <0.001 ***, 0.818
*J*_b_ (F, *p*, η^2^)	0.810, 0.599, 0.178	21.716, <0.001 ***, 0.591	27.503, <0.001 ***, 0.786
*J*_m_ (F, *p*, η^2^)	1.495, 0.201, 0.285	23.016, <0.001 ***, 0.605	29.206, <0.001 ***, 0.796

Note: F = analysis of variance test statistic; *p* = significance level; η^2^ = partial eta squared (effect size). The significance threshold was set at *p* < 0.05. Asterisks indicate the level of significance: *p* < 0.05 (*), *p* < 0.01 (**), and *p* < 0.001 (***), and *p* ≥ 0.05 denotes not significant.

**Table 10:** Results of post hoc tests for factor A.

Comparison	*G*_F_	*J*_IC_	*J*_IF_	*J*_c_	*J*_b_	*J*_m_
R0–R25	0.005	0.723	0.233	0.008	0.004	0.044
R0–R50	<0.001	0.024	<0.001	<0.001	<0.001	<0.001
R25–R50	0.001	0.125	0.003	0.001	0.011	<0.001

**Table 11:** Results of post hoc tests for factor B.

Comparison	*G*_F_	*J*_IC_	*J*_IF_	*J*_c_	*J*_b_	*J*_m_
0–30 °C	0.089	0.943	0.156	0.113	0.291	0.098
0–70 °C	<0.001	0.990	0.003	0.000	0.000	0.000
0–100 °C	0.007	0.474	0.344	0.008	0.010	0.030
0–150 °C	<0.001	0.050	0.010	0.000	0.001	0.001
30–70 °C	0.052	0.743	0.475	0.035	0.066	0.059
30–100 °C	0.819	0.141	0.990	0.792	0.533	0.983
30–150 °C	<0.001	0.008	0.000	0.000	0.000	0.000
70–100 °C	0.389	0.757	0.238	0.322	0.745	0.176
70–150 °C	<0.001	0.133	0.000	0.000	0.000	0.000
100–150 °C	<0.001	0.725	0.000	0.000	0.000	0.000

## Data Availability

The original contributions presented in this study are included in the article. Further inquiries can be directed to the corresponding author(s).
